# Effects of alternating heat and cold stimulation using a wearable thermo-device on subjective and objective shoulder stiffness

**DOI:** 10.1186/s40101-021-00275-9

**Published:** 2022-01-03

**Authors:** Tomonori Sawada, Hiroki Okawara, Daisuke Nakashima, Shuhei Iwabuchi, Morio Matsumoto, Masaya Nakamura, Takeo Nagura

**Affiliations:** 1 Diagnosis and Treatment Division, Nagura Orthopedic Clinic, Chuo, Tokyo, Japan; 2 Department of Orthopaedic Surgery, Keio University School of Medicine, Shinjuku, Tokyo, Japan; 3 Department of Clinical Biomechanics, Keio University School of Medicine, Shinjuku, Tokyo, Japan

**Keywords:** Alternating heat and cold stimulation, Muscle hardness, Trapezius muscle, Skin temperature

## Abstract

**Background:**

Technological innovations have allowed the use of miniature apparatus that can easily control and program heat and cold stimulations using Peltier elements. The wearable thermo-device has a potential to be applied to conventional contrast bath therapy. This study aimed to examine the effects of alternating heat and cold stimulation (HC) using a wearable thermo-device on subjective and objective improvement of shoulder stiffness.

**Methods:**

Twenty healthy young male individuals (20.3 ± 0.6 years) participated in this study. The interventions were randomly conducted under four conditions, including HC, heat stimulation, cold stimulation, and no stimulation on their bilateral trapezius muscle, after a 30-min typing task. Each intervention was administered at least 1 week apart. The analyzed limb was the dominant arm. Muscle hardness was assessed using a portable muscle hardness meter, as well as the skin temperature over the stimulated area. After each condition, the participants were asked for feedback regarding subjective improvement in refreshed feelings, muscle stiffness, and muscle fatigue using an 11-point numerical rating scale.

**Results:**

With regard to muscle hardness, only the HC condition significantly decreased from 1.43 N to 1.37 N (*d* = 0.44, *p* < 0.05). Additionally, reduced muscle hardness in HC condition was associated with the degree of skin cooling during the intervention (cold max: *r* = 0.634, *p* < 0.01; cold change: *r* = −0.548, *p* < 0.05). Subjective improvement in refreshed feelings, muscle stiffness, and muscle fatigue was determined in the HC and heat stimulation conditions compared with the no stimulation condition (*p* < 0.01 and *p* < 0.05, respectively). Moreover, the HC condition showed significantly greater improvements in muscle stiffness and fatigue compared to the cold stimulation condition (*p* < 0.05).

**Conclusions:**

The current study demonstrated that HC promoted not only better subjective symptoms, such as muscle stiffness and fatigue, but also lesser muscle hardness. Furthermore, an association was observed between the degree of skin temperature cooling and reduced muscle hardness during HC. Further investigations on the ratio and intensity of cooling should be conducted in the future to establish the optimal HC protocol for muscle stiffness or fatigue.

**Trial registration:**

UMIN000040620. Registered 1 June 2020

## Background

Increased computer usage has been linked to a high prevalence of musculoskeletal symptoms in the neck and upper extremities [1–3], with estimates in 2010 showing a global neck pain prevalence of approximately 332 million people (4.9% of the population) [4, 5]. Desk workers engaged in intensive and prolonged computer use have been known to be at a higher risk for having neck to shoulder pain [6–8]. In addition, previous studies have shown that patients with neck or shoulder pain tend to have stiff trapezius muscles [9, 10]. Therefore, increased trapezius muscle stiffness is believed to be caused by sustained low-intensity muscle contraction due to repetitive tasks, such as desk work, and pain may be caused by local ischemia and accumulation of metabolic products [3, 11]. Although the recent COVID-19 pandemic has reduced sociality through immobility, the amount of time spent using smartphones and social media has likely increased due to the “stay at home order” [12]. On the contrary, no firm solutions that address work-related neck and shoulder stiffness and pain have been available. Therefore, establishing a safe, noninvasive, and scientific self-management method for this condition warrants urgent attention.

Bathing has long been commonly and widely used in health preservation and rehabilitation [13, 14]. Contrast baths, one of the bathing methods wherein alternating hot and cold water is applied, have been thought to cause intermittent vasoconstriction and vasodilation that induce a vascular pumping effect, consequently promoting increased tissue blood flow and oxygenation that improve healing; enhances tissue waste-product transportation, which reduce edema; improve limb function; and promote a quicker recovery [15]. Therefore, contrast bath therapy is believed to be more efficient in improving circulation than a simple hot whirlpool and has been frequently and widely used for athletes, in particular, for rapid recovery after games or pain relief following acute and sub-acute soft tissue injuries [16, 17]. In contrast, the aforementioned therapy is limited by the need for large baths, difficulty with water temperature control given that it changes with each immersion, and hygiene problems when multiple people use the same baths. Additionally, considering that the temperature settings, number of sets, and duration of hot and cold water application vary from one study to another, clear evidence has yet to be established [18–21].

Technological innovations have allowed the use of miniature apparatus that can easily control and program heat and cold stimulations using Peltier elements. Such a concept apparatus can provide rapid and quantitative localized heating and cooling stimulation at increments of 0.1°, enabling optimal temperature protocol management to prevent complications, such as hot/cold burns, and to produce effects; it can be widely used as an alternative to conventional contrast bath therapy. Another advantage is that it more quantitatively verifies the effects of temperature changes. However, a recent report has shown that alternating heat and cold stimulation (HC) with an infrared device and a cryotherapy device can improve muscle stiffness and pain to the same degree as the conventional contrast bath therapy [22]. Although the use of infrared and cryotherapy devices is more efficient in terms of mobility and temperature maintenance compared with conventional contrast bath therapy, the new device using Peltier elements is more versatile because it can provide HC in one small device. Thus, the current study aimed to examine the effects of HC using the new apparatus on subjective improvement in shoulder muscle stiffness and/or fatigue and objective improvement in shoulder muscle hardness. We hypothesized that HC would produce better improvements than heat or cold stimulation alone. The results of this study will help establish an effective self-management method using HC for shoulder muscle stiffness or fatigue, which will help maintain the health of many desk workers.

## Methods

### Participants

After recruiting participants from one hospital and one university in the autumn of 2020, twenty healthy young male individuals without any orthopedic abnormalities of the neck and shoulders were included in this study (Table 1). A questionnaire was used to record average daily smartphone use and typing time over a recent month. All participants were informed regarding the purpose of this study, after which informed consent was obtained prior to participation. The experimental design was approved by the P-One Clinic Ethical Committee. This study was conducted in accordance with the Declaration of Helsinki.

**Table 1:** Participant characteristics (*n* = 20)

Age (years)	20.3 ± 0.6
Height (m)	1.74 ± 0.1
Weight (kg)	71.0 ± 12.2
BMI (kg/m^2^)	23.4 ± 3.0
Dominant hand (*n*)	Right: 19, Left: 1
Time using smartphone per day (h)	5.4 ± 2.7
Time of typing per day (h)	1.8 ± 1.6

Values are mean ± SD

### Experimental protocol

All participants were asked to visit our laboratory four times throughout the study to compare the four intervention conditions. The room temperature within the laboratory was set between 24 and 26 °C. The interventions were randomly conducted under the following four conditions: alternating heat and cold stimulation (HC), heat stimulation (HEAT), cold stimulation (COLD), and no stimulation (NO). Each intervention was administered at least 1 week apart. The flow of each intervention day was kept identical, with each of the interventions being conducted after the typing task. The interventions were performed on the bilateral upper trapezius muscles, which had been reported to be most commonly affected with myofascial trigger points [23, 24]. The analyzed limb was the dominant arm. Firstly, trapezius muscle hardness was assessed, after which 30-min typing task was performed to induce fatigue around the shoulder according to previous studies [25, 26]. Specifically, dual desktop monitors with 20-inch screens (Dell P2011H; DELL Inc., TX, USA) and a keyboard were placed at the center of a standard computer desk, while the typing text and a commercially available document entry software (Word; Microsoft Corporation, WA, USA) were displayed on right and left side of the dual monitors, respectively. The angle between the dual monitors was set to face inward at 30°. The pointing device was a mouse, allowed to be used mainly when scrolling the screen. Thereafter, we instructed the participants to transcribe as much of the text as possible into the document entry software. During the typing task, the chair height was adjusted and the participants were instructed to remain seated with their knees at 90° and forearms on the desk and to continue typing in the same position as much as possible. The texts used for typing task comprised four out-of-copyright Japanese novels, which were randomly displayed to avoid duplication. After the typing task, muscle hardness was re-assessed and followed by one of the four interventions. After the intervention, muscle hardness was re-assessed, and participants were asked to rate the degree to which they felt in terms of (1) refreshed feeling, (2) improved muscle stiffness, and (3) improved muscle fatigue in the area of intervention using an 11-point numerical rating scale (0 to 10, 0 = not at all, 10 = very much), which had also been utilized to assess except for pain [27].

### Intervention

**Fig. 1:**
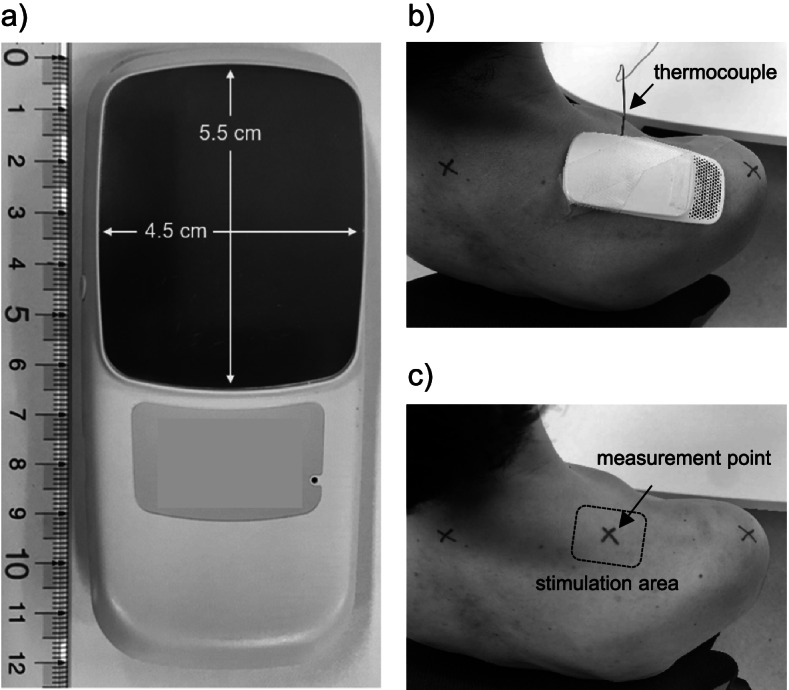
Prototype of the wearable thermo-device (WTD) used in this study. (**a**) The stimulation area that cools/heats the skin surface is approximately 4.5 × 5.5 cm. (**b**) Intervention scene using WTD. A thermocouple (solid arrows) was set up to measure the skin temperature during the intervention. (**c**) The measurement point of trapezius muscle hardness and skin temperature (solid arrow) and the stimulation area of WTD (dotted area)

**Fig. 2:**
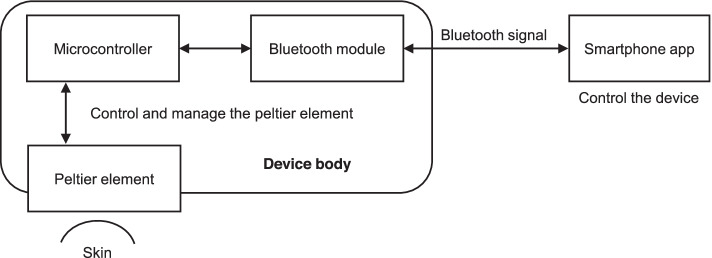
Schematic diagram of the wearable thermo-device (WTD)

The current study performed heat and/or cold stimulation using a prototype of a wearable thermo-device (hereinafter referred to as WTD; Sony Corporation, Tokyo, Japan) (Fig. 1a). WTD is a product containing a Peltier element that can provide heat and cold temperatures. The Peltier element functions through heat transfer via voltage application, which allows for product surface cooling or heating. Thus, WTD can cool or heat the surface that comes into contact with the skin (called the cold/heat part). The cooled/heated area is approximately 4.5 × 5.5 cm. WTD can be operated via a dedicated smartphone application provided by Sony Corporation, through which repeated cooling, heating, and stopping at a fixed number of seconds are possible (Fig. 2), a function utilized herein. WTD was taped to the skin of the upper trapezius muscle to randomly administer the aforementioned four stimulation conditions (i.e., HC, HEAT, COLD, and NO condition) (Fig. 1b). In the HC condition, heat stimulation was given for 3 min, cold stimulation for 1 min [15], and movement cessation between heat and cold stimulation for 10 s. The 10-s cessation time was set to achieve stable quality by considering the thermal stress strain that occurs inside the module during rapid cycling of heating and cooling. Therefore, five sets of the HC condition would take a total of 22 min, whereas the HEAT and COLD conditions would take 20 min. Under the three stimulation conditions, the intensity of heat and cold stimulations was the same. More specifically, the maximum and minimum temperatures at the stimulation area of the device when continuously heated or cooled at this intensity for 20 min were 42.2 °C ± 0.4 °C and 24.1 °C ± 1.0 °C, respectively. In the NO condition, WTD was set up for 20 min with no power. During stimulation, participants rested in a relaxed position in a chair with a backrest.

### Measurements

The muscle hardness and temperature of the skin above the trapezius muscle were assessed. The measurement point was set at the midpoint between the seventh cervical spinous process and the tip of the acromion [25] (Fig. 1c). Muscle hardness was qualitatively evaluated using a portable muscle hardness meter (NEUTONE TDM-Z2; TRY-ALL Corp., Chiba, Japan) by a trained examiner (TS) who was blinded to which interventions conducted. A similar device had been used in previous studies [28, 29]. Our previous study showed that the muscle hardness meter had excellent intra-tester reliability for the trapezius muscle (ICC_1, 5_ = 0.992–0.995) [30]. Given that the values measured using this device have no units (displayed between 0 and 100), they were converted to Newton using the following formula: *N* = 0.023 × measured value + 0.532 based on the manufacturer’s report. This was performed five times at each measurement time, with the mean value of the five trials being used as the muscle hardness value. Muscle hardness was assessed thrice: at baseline, after the typing task, and after the intervention.

**Fig. 3:**
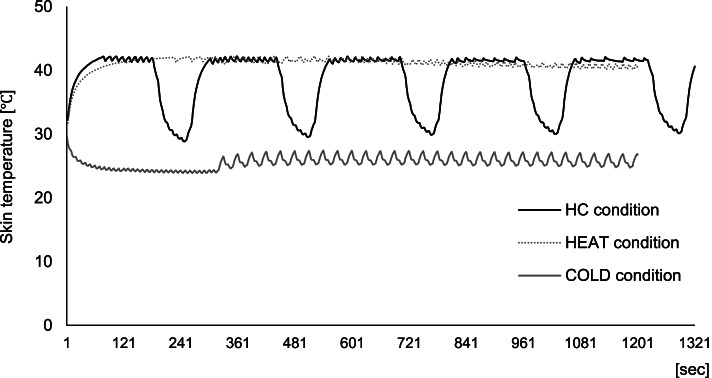
A representative example of skin temperature on the trapezius muscle for each condition: HC, alternating heat and cold stimulation; HEAT, heat stimulation; COLD, cold stimulation

Additionally, during each intervention, the skin temperature of the stimulation area was measured using a thermocouple (JBS-7115-5M-T; GRAPHTEC Corporation, Yokohama, Japan) and the obtained data were continuously recorded at 1 Hz in a data logger (midi LOGGER GL840; GRAPHTEC Corporation, Yokohama, Japan) (Fig. 3). The skin temperature at the start of the intervention was used as the baseline. The maximum heating (heat max) and cooling (cold max) temperatures from baseline were then determined. Furthermore, cumulative changes in skin temperature, that is, the timed integral of change in skin temperature from baseline, was calculated for intervention duration and defined as “heat and cold changes,” respectively.

### Statistical analysis

Although the present study performed the interventions on the bilateral trapezius muscles, analysis for muscle hardness and skin temperature was performed on the dominant side. Initially, differences in muscle hardness before and after the typing task and after the typing task and intervention were compared for each condition. The effect size of each comparison was also calculated based on Cohen’s *d* [31]. Moreover, a post hoc power analysis using G*Power 3.1.9 was used. Upon determining that the intervention promoted improvements in muscle hardness, correlations between changes in muscle hardness (post-intervention value minus post-typing value) and skin temperature trends during the intervention were calculated using Pearson’s product-moment correlation coefficient. Lastly, differences in the degree of subjective improvement among the four conditions were compared using the Freidman test. All statistical analyses were performed using SPSS Statistics version 25.0 (IBM Corp., Armonk, NY, USA), with statistical significance being set at 0.05.

## Results

### Changes in muscle hardness

Table 2 summarizes the mean values for trapezius muscle hardness before typing, after typing, and after intervention in the four conditions. Contrary to our expectation, even after a 30-min typing task, no significant difference in muscle hardness was found for all conditions. However, the muscle hardness in the HC condition showed a significant decrease to 1.37 N (*d* = 0.44, *Power* = 0.36, *p* < 0.05) after the intervention, suggesting that the trapezius muscle became softer after the typing task. In the other three conditions, no significant differences in muscle hardness values were noted after the typing task and intervention.

**Table 2:** Muscle hardness values before typing, after typing, and after the intervention according to the different conditions (*n* = 20)

Conditions	Baseline (*N*)	After typing (*N*)	After the intervention (*N*)
HC	1.41 ± 0.15	1.43 ± 0.13	1.37 ± 0.14*
HEAT	1.43 ± 0.18	1.46 ± 0.16	1.44 ± 0.15
COLD	1.39 ± 0.19	1.44 ± 0.18	1.44 ± 0.18
NO	1.41 ± 0.16	1.42 ± 0.18	1.43 ± 0.17

*HC* alternating heat and cold stimulation, *HEAT* heat stimulation, *COLD* cold stimulation, *NO* no stimulation

*p < 0.05 (versus after typing)

### Relationship between changes in muscle hardness and skin temperature during intervention

**Fig. 4:**
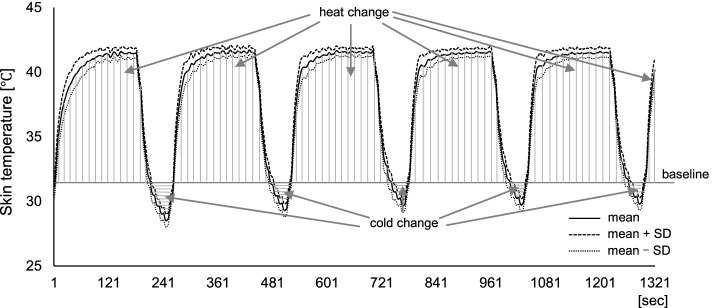
Changes in skin temperature over the trapezius muscle of the dominant arm under the HC condition (*n* = 19). The solid line shows the mean value, while the dotted lines show the mean ± standard deviation. The timed integral of the change in skin temperature from baseline was calculated for intervention duration and defined as heat change (sum of the vertical line area) and cold change (sum of the horizontal line area)

**Fig. 5:**
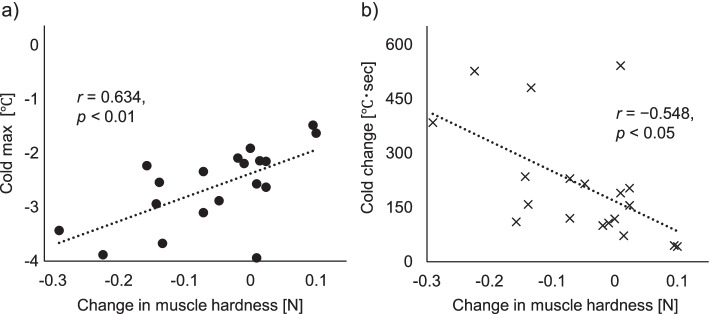
Scatter diagram representing the changes in muscle hardness and skin temperature on the trapezius muscle under HC conditions: (**a**) “Cold max” was defined as the maximum cooling temperature from baseline (i.e., the skin temperature at the start of the intervention) during the intervention. (**b**) “Cold change” was defined as the cumulative cooling change in skin temperature during the intervention duration, which was calculated as the time integral of the cooling temperature change from baseline

Considering our results showing an improvement in muscle hardness under the HC condition, the association between changes in skin temperature during the HC condition and muscle hardness was investigated. Figure 4 depicts the trend in skin temperature on the trapezius muscle during stimulation in the HC condition (*n* = 19, one participant was excluded due to incomplete skin temperature data). The average baseline skin temperature was 31.1 °C ± 0.8 °C, with the intervention resulting in a maximum of 41.7 °C ± 1.0 °C and a minimum of 28.5 °C ± 0.3 °C, respectively. Furthermore, correlation analysis showed that changes in muscle hardness after the intervention were significantly associated with the degree of skin cooling during the intervention (Fig. 5, cold max: *r* = 0.634, *p* < 0.01; cold change: *r* = −0.548, *p* < 0.05). However, no significant association between changes in muscle hardness and degree of skin heating was observed (heat max: *r* = 0.212, *p* = 0.384; heat change: *r* = 0.222, *p* = 0.362).

### Subjective improvement

**Fig. 6:**
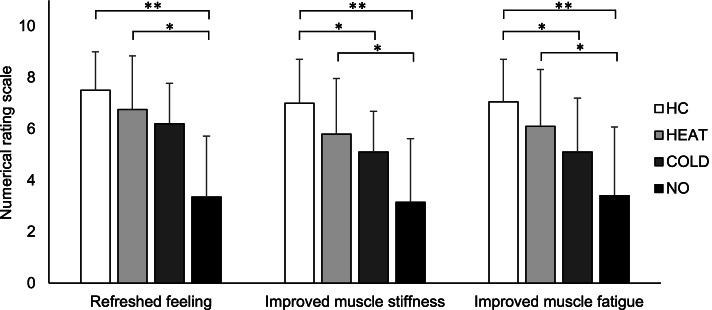
Subjective values (refreshed feeling, improved muscle stiffness, and improved muscle fatigue in the area of intervention) based on the 11-point numerical rating scale (0 to 10, 0 = not at all, 10 = very much) after the four interventions: HC, alternating heat and cold stimulation; HEAT, heat stimulation; COLD, cold stimulation; NO, no stimulation. *p < 0.05, **p <0.01

Significant differences in all three subjective improvement items were observed between interventions (Fig. 6). Multiple comparison analysis showed that HC and HEAT conditions promoted significantly greater refreshed feelings than the NO condition. Moreover, HC and HEAT conditions showed significantly greater improvements in muscle stiffness and fatigue compared to the NO condition, whereas the HC condition showed significantly greater improvements compared with the COLD condition. In all conditions, no cases had worsening muscle stiffness or fatigue.

## Discussion

The results presented no change in muscle hardness had been observed in the HEAT and COLD conditions alone, muscle hardness after the HC intervention was lesser than that after the typing task. In addition, improvements in muscle hardness in the HC condition were found to be associated with the degree of skin cooling during the intervention. Furthermore, subjective improvements in refreshed feelings, muscle stiffness, and muscle fatigue were observed in HC and HEAT conditions.

Participants in the present study were instructed to perform a 30-min typing task to activate the trapezius muscle, one of the common muscles in which complaints of pain and muscle tension occurs among desk workers [6, 7], based on previous studies [25, 26]. Notably, no significant difference in trapezius muscle hardness had been observed before and after the typing task. Horikawa [25], who examined changes in trapezius muscle hardness after a typing task in healthy young subjects, demonstrated an increase in muscle hardness depending on the screen setting environment. Specifically, he reported no change in the muscle hardness after 30 min of typing when the upper edge of the display was set at an appropriate height (5–10° below eye level) in relation to the subject’s eye level. However, when the display was too high (15–20° above eye level) or too low (15–20° below eye level) or when typing on a notebook computer (20–25° below eye level), he noted an increase in muscle hardness. In addition, Goostrey et al. [32] examined the differences in neck movement and trapezius muscle activity depending on the position while typing a document and reported that the neck flexion and trapezius muscle activity was greatly increased when the document was placed on the desktop. Therefore, the lack of an obvious increase in muscle hardness following the typing task performed in the present study could be attributed to the fact that the participants were close to the ideal typing posture, suggesting that a typing task with a poor posture should be further examined.

In this study, only the HC condition promoted decreased muscle hardness, an objective measure. Thermotherapy is generally expected to increase tissue temperature, increase local blood flow, increase soft tissue extensibility, cause local vasodilation, increase metabolite production, and reduce muscle spasm [15, 33, 34]. Therefore, decreased muscle hardness was expected in the HEAT condition; however, as expected, no significant decrease in muscle hardness was observed in the HEAT condition. One possible reason for this is that the stimulation area was relatively small. In general, the threshold of skin temperature sensation is affected by the rate of temperature changes and stimulated area [35], that is, the larger the stimulation area, the greater the effect. Therefore, although the WTD used in this study sufficiency increased the skin surface temperature, the stimulation area was small, and the effect may have been limited. However, it is very interesting to note that the combination of the same intensity stimulation as HEAT and COLD conditions produced statistically significant changes in the soft-tissues stiffness, including that of the muscles. Currently, the effectiveness of contrast bath therapy for patients with neck or shoulder pain has not been established, and only a few studies have shown the effects of HC [22]. Although we could not demonstrate the extent of HC effectiveness for muscles with pain, HC using the WTD resulted in an improvement in trapezius muscle hardness, which is a risk factor for neck or shoulder pain. As the WTD can be easily used anytime and anywhere, we believe it can be useful for self-management of desk workers who often need to work on the PC for a long time.

More interestingly, our results showed that the reduction in muscle hardness during HC was associated with the degree of skin temperature cooling rather than heating. This result indicates the involvement of physiological effects expected with cold stimulation. During rehabilitation, cold therapy has been used to control inflammation, pain, and edema and reduce spasticity [15, 34, 36]. Given that participants included herein were unlikely to have edema and pain due to acute muscle tissue inflammation, reduced spasticity could have been primarily due to reduced muscle stiffness. Local application of cold is used clinically to diminish spastic muscle resistance to rapid stretching, to reduce clonus, and to increase range of motion [37]. Previous studies have shown that cold therapy reduces the stretch reflex of the Achilles tendon following muscle cooling [38, 39]. Considering that the expected improvements in peripheral blood flow following superficial heat stimulation is limited to muscles located deeper within the skin and subcutaneous tissue [34], the combination of cold stimulation may promote further muscles relaxation through the nervous system.

The results of the present study also showed that the HC and HEAT conditions promoted greater improvements in subjective symptoms than the NO condition. Moreover, the HC condition showed significantly greater improvements in muscle stiffness and fatigue than the COLD condition. In contrast, the COLD condition did not promote significantly greater improvements in subjective symptoms as compared to the no stimulus condition. A previous study of contrast bath therapy generally used a hot and cold ratio of 4:1 or 3:1, with a larger time ratio for hot water immersion [15]. This may therefore suggest that heat stimulation had a stronger impact on subjective symptom improvement than cold stimulation, particularly on muscle stiffness and fatigue. Fatigue is defined as the sensation of tiredness and the associated decrease in muscular performance and function [40]. The subjective symptoms of muscle disorders are constant; muscle fatigue and stiffness are consistent with radiating pain [11]. Repetitive fatigue-causing work is closely associated with work-related musculoskeletal disorders [41], which may be influenced by prolonged static work postures [42]. The muscle has been reported to become harder in pathological conditions, such as muscular damage, spasms, cramps, and edema [43–45]. Therefore, muscle hardness evaluation is considered useful to assess muscle fatigue associated with sustained muscle contraction. Generally, one of the main purposes of thermotherapy in rehabilitation is to increase soft tissue extensibility [34]. In a previous in vitro study, Lehmann et al. immersed the explanted rat tendons in 45 °C warm water and reported increased extensibility [46]. In fact, several studies report on the use of thermotherapy to increase a joint motion range by increasing the extensibility of soft tissues, such as tendons, joints, ligaments, and skeletal muscles [47]. Our results may indicate that if the heat and cold ratio is 3:1, soft tissue extensibility can be improved to as much as with heat stimulation without the negative effect of cold stimulation.

Some limitations of the current study warrant discussion. First, as only healthy young (range, 19–22 years) male individuals were included in this study, its results could not be generalized on the whole population including women, and the relationship between pain and muscle hardness could not be validated. In particular, as previous studies have shown that individuals with neck or shoulder pain tend to have stiff trapezius muscles [9, 10], they may have achieved greater improvement in muscle hardness with the heating/cooling intervention performed in this study. Second, given that only trapezius muscle hardness had been measured, the effects of HC on other muscles associated with chronic neck and shoulder pain (i.e., the levator scapulae and neck extensors, such as the splenius cervicis muscles) remain unknown. Third, although HC and HEAT promoted an overall refreshed feeling, the detailed mechanisms for such improvement of symptoms remain unknown. Future research assessing autonomic nervous system activity using heart rate variability analysis may therefore provide new insights.

## Conclusions

By employing strict temperature control using Peltier elements, the current study demonstrated that HC not only improved subjective symptoms such as muscle stiffness and fatigue but also decreased muscle hardness. Furthermore, a relationship between the degree of the skin temperature cooling and the decreased in muscle hardness was observed during HC. Further investigations on the ratio and intensity of cooling should be conducted in the future to establish the optimal HC protocol, while elucidating the muscle stiffness mechanism or fatigue improvement.

## Data Availability

The datasets used and/or analyzed during the current study are available from the corresponding author on reasonable request.
